# Comparison of Midazolam and Propofol for BIS-Guided Sedation During Regional Anaesthesia

**Published:** 2009-12

**Authors:** Priyanka Khurana, Ankit Agarwal, RK Verma, PK Gupta

**Affiliations:** 1 Ex.P.G.Student, Deptt. Of Anaesthesiology, BHU, Varanasi; 2 Sr.Resident, Deptt. Of Anaesthesiology, BHU, Varanasi; 3 Reader, Deptt. Of Anaesthesiology, BHU, Varanasi; 4 Professor, Deptt. Of Anaesthesiology, BHU, Varanasi

**Keywords:** Propofol, Midazolam, Sedation, BIS

## Abstract

**Summary:**

Regional anaesthesia has become an important anaesthetic technique. Effective sedation is an essential for regional techniques too. This study compares midazolam and propofol in terms of onset & recovery from sedation, dosage and side effects of both the drugs using Bispectral Index monitoring. Ninety eight patients were randomly divided into two groups,one group recieved midazolam infusion while the other recieved propofol infusion until BIS reached 75. We observed Time to reach desired sedation, HR, MABP, time for recovery, dose to reach sedation and for maintenance of sedation and side effects if any. The time to reach required sedation was 11 min in Midazolam group(Group I) while it was 6 min in Propofol group(Group II) (p=0.0). Fall in MABP was greater with propofol. Recovery in with midazolam was slower than with propofol (18.6 ± 6.5 vs 10.10±3.65 min) (p=0.00). We concluded that both midazolam and propofol are effective sedatives, but onset and offset was quicker with propofol, while midazolam was more cardiostable.

## Introduction

In the recent days regional techniques have come to take an upper hand in anaesthesia over general anaesthesia owing to its certain, often underestimated advantages such as lesser chances of airway compromise and aspiration, facilitation of postoperative analgesia, inherent benefit in some preexisting medical conditions and avoidance of operation theatre pollution. The concept of Monitored Anaesthesia Care has come to highlight the fact that a vigil on patient's vitals and monitoring of various aspects of regional anaesthesia are as important as in general anaesthesia^1^.

Amongst the armamenterium of monitoring equipment available to the modern anaesthetist, BIS is perhaps the latest and the best suited tool.^2^ Besides providing an idea about the hypnotic state of the patient, it also enables titration of anaesthetic agents so as to avoid adverse effects as awareness due to inappropriate dosage as well as unwanted effects of overdosage.

We performed a study comparing sedative effects of propofol and midazolam using BIS in regional anaesthesia. Although literature is flooded with reports on use of BIS during general anaesthesia, it was still deficient in studies involving regional anaesthesia. We therefore evaluated BIS while under sedation. Propofol and midazolam both are established sedative agents both intraoperatively and in an ICU^3^,^4^.

The aim of our study was to find out the time for onset and recovery from sedation with both drugs, using BIS as a standard measure of depth of sedation and to evaluate and compare the properties of propofol and midazolam in terms of haemodynamics, side effects and dosage requirement as adjuncts to spinal anaesthesia.

## Methods

The study was conducted in 98 ASA grade I and II patients between age 20-50 years undergoing lower abdominal, perineal and lower limb surgeries under age as well as unwanted effects of overdosage. combined spinal epidural block upto T_10_ level.

Patients were randomly allocated to one of the following two groups:

Group I- (n=50) Midazolam 0.1% infusion starting with 0.5 mg.kg^−1^.h^−1^ till BIS level reached 75 and then dose reduced and titrated to maintain a BIS of 65-85.

Group II- (n=48) Propofol 1% infusion starting with 6mg.kg^−1^.h^−1^ till BIS level reached 75 and then dose was reduced and titrated to maintain a BIS of 65-85.

A written informed consent was taken from all patients. They were fasted for a minimum of 6 hours before surgery. No preoperative opioids or prophylactic antiemetics were given. No other preoperative medication was allowed. Patients suffering from heart disease, hypertension, diabetes, spinal deformity, neurological problem or any bleeding disorder were excluded from the study. All patients were monitored with an electrocardiograph, noninvasive blood pressure, pulse oximeter and BIS monitor. Baseline readings were recorded. Preloading was done with 15ml.kg^−1^ of Ringer lactate prior to block. Combined spinal and epidural block was given. Epidural catheter was put at L3-4 or L2-3 level, 3 ml of 0.5% bupivacaine was given into the subarachnoid space and the epidural catheter was maintained for providing postoperative analgesia. 1 % propofol or 0.1% midazolam infusion was started with the help of a manually controlled variable rate infusion pump.

Propofol infusion was given at a rate of 6mg.kg^−1^.hr^−1^ and midazolam at 0.5 mg.kg^−1^.hr^−1^ and after reaching a BIS value of 75, the rate of infusion was reduced to half and then with subsequent observations, the anaesthetics were titrated to keep a BIS level between 65 and 85.

Blood pressure, heart rate, oxygen saturation, and BIS level were assessed every 2 min till maintenance dose was reached ie a BIS level of 65-85 and then every 10 min till 105 min or till the end of surgery which- ever was earlier and every 15 min (if duration of surgery extended beyond that).

O_2_ inhalation by ventimask was given when SpO_2_ came down below 90% and vasopressor was given if MAP decreased beyond 20% of baseline.

Following observations were made:

1. Time to reach required level of sedation.
2. Duration of surgery
3. Duration of infusion
4. HR, Mean Arterial pressure, arterial saturation of oxygen were recorded every 2 minutes till required sedation level was reached and then every 10 min till 105 min or end of the surgery whichever was earlier and then every 15 minutes.
5. Time taken for recovery (for comparison, BIS>90 was taken as a recovery parameter )
6. Side effects
  - Awareness
  - Nausea & Vomiting
  - Pain in arm
7. Dose to reach required level of sedation.
8. Dose to maintain required level of sedation

Statistical analysis was done with independent t test for age, weight, duration of surgery and end infusion, time for recovery, heart rate, mean arterial pressure, and SpO_2_ at various intervals. Chi square test was applied for sex distribution and for adverse effects as respiratory obstruction, apnea, laryngospasm, nausea & vomiting, awareness, hypotension, and oxygen supplementation. Paired t test was applied for intra-group variation in heart rate and mean arterial pressure. Fischer's exact test was used for incidence of complications.

## Results

The mean age, sex and body weight in the two groups were statistically similar. The mean of various time intervals in the two groups is as shown in Table 1. The mean time to reach the required level of sedation in group I was 11.0 ± 0.5 minutes which was about 5 minutes later than in group II (6.2 ± 0.2min) (p=0.00), thus the difference in mean time to reach required sedation level was statistically highly significant. The difference in mean duration of infusion in the two groups was statistically insignificant (73.2 ± 23.9 vs 71.1 ± 18.0) (p=0.50). The time for recovery in group I (midazolam group) was more than in group II (propofol group) (18.6 ± 6.5 vs 10.10 ± 3.65 min) (p=0.00) and it was highly significant.

**Table 1:** Demography of the patients

	Group I Midazolam (n=50)	Group II Propofol (n=48)	p value
Age (years)	42.6±6.31	39.5±6.49	0.34
Sex (M/F)	16/34	18/30	0.54
Duration of surgery (min)	66.9±22.7	64.9±17.87	0.633
Initial dose of the drug to achieve target BIS (mg/kg/h)	0.5	6.0	
Mean rate of infusion during maintenance (mg/kg/h)	0.1±0.38	2.2±0.56	
Time to reach required level sedation (min)	11.0±3.66	6.2±1.88	0.00
Time taken for recovery (min)	18.6±6.50	10.1±3.65	0.00

data are mean ± SD or n

The mean ± SD of heart rates at various time intervals is shown in Table 2. In Group I, the initial mean heart rate was 86.0 ± 11.9 which gradually decreased to 78.0 ± 10.6 at 25 min while in Group II initial mean heart rate of 85.37 ± 11.97 per min which gradually decreased to 75.6 ± 12.3 at 25 min. In between group comparison of mean heart rate at various time intervals using independent t test in the two groups revealed the difference was not significant at almost all time intervals. However, the comparison of heart rate at various time interval with the baseline within the same group using t test showed significant difference at various time intervals

**Table 2:** Comparison of heart rate(bpm) in both groups at various time intervals

Time interval(min)	Group I	Group II	p value
0	86.8±11.97	85.3±11.97	0.557
2	85.7±14.23	81.3±11.92++	0.105
4	84.7±15.77+	79.0±12.22++	0.051
6	79.1±18.82++	75.6±12.71++	0.292
8	79.9±16.10++	77.4±12.69++	0.404
10	79.4±14.28++	77.7±13.29++	0.546
12	77.8±12.97++	77.9±12.69++	0.945
14	78.4±12.20++	79.7±15.18++	0.633
16	78.8±10.86++	80.9±17.64+	0.482
25	78.6±10.69++	75.6±12.36++	0.199
35	77.9±10.84++	76.1±13.14++	0.462
45	79.4±10.10++	77.0±15.36++	0.379
55	82.5±8.83+	82.1±13.62+	0.856
65	85.4±7.38	80.2±K59++	0.085
75	86.0±9.06	79.5±17.35++	0.224
85	86.3±10.76	85.5±14.15	0.880
95	88.0±8.60	101.5±2.08	0.014
105	83.7±9.97	96.0±1.41	0.178
120	79.5±16.26	96.5±3.53	0.285

++ p value <0.01(HS within group)

+ p value <0.05 (Significant within group)

Mean arterial pressures: The mean ± SD of MAP values are shown in Table 3. The mean arterial pressure in Group I initially was 81.7 ± 6.8 mmHg which gradually decreased to 76.8 ± 6.9 mm Hg while, in Group II mean MAP at baseline was 83.1 ± 8.5 mm Hg which gradually decreased to 68.25 ± 2.98 mm Hg.

**Table 3:** Comparison of MABP(mmHg) in both groups at various time intervals

Time interval	Group I	Group II	p value
0	81.7±6.82	83.1±8.54	0.363
2	79.7±6.08++	79.0±5.97++	0.556
4	77.6±6.02++	77.4±6.52++	0.848
6	76.9±6.3++	76.4±6.84++	0.707
8	76.8±6.97++	75.7±6.47++	0.443
10	77.5±7.53++	75.1±6.09++	0.085
12	76.7±8.19++	74.8±6.43++	0.214
14	76.1±9.38++	74.4±6.41++	0.304
16	75.9±9.93++	74.4±6.58++	0.314
25	76.0±9.55++	73.7±7.48++	0.183
35	75.7±10.96++	72.6±7.79++	0.105
45	78.7±10.53+	71.3±6.77++	0.000
55	75.7±10.41++	71.1±7.28++	0.020
65	75.3±9.83++	68.9±6.85++	0.084
75	82.3±9.84	68.9±8.34+	0.000
85	84.6±10.52	68.3±7.37+	0.000
95	82.1±10.49	68.2±2.98+	0.032
105	78.2±13.52	71.0±4.76	0.351
120	69.0±12.72	75.5±0.70	0.546

++ p value <0.01(HS within group)

+ p value <0.05 (Significant within group)

Between-group comparison showed that the difference was not significant up to 35 min, but it become significant at 45,55,75,85 & 95 min to become non-significant again at105 and 120 min.

The incidence of complications is as shown in Table 4.

**Table 4:** Incidence of complications

	Restlessness	Pain in arm	Nausea & Vomiting	Awareness
Group I	4 (8 %)	0 (0 %)	8 (16 %)	10 (20 %)
(n=50)				
Group II	7 (14.56 %)	3 (6.25 %)	4 (8.3 %)	8 (16.7 %)
(n=48)				
p value	0.24	0.11	0.40	0.86

Hypotension defined a decrease of MAP > 20% from baseline was seen in 8 (16%) cases in Group I while in group II, it was seen in 13 cases (27.1%); the difference was not significant.

To maintain the desired sedation ie BIS 65-75, maintenance dose of 2.2 ±0.5 mg/kg/h propofol and 0.12±0.38 mg/kg/h for midazolam had to be given in the two groups respectively.

Oxygen supplementation: It was provided when SpO2 level was below 90. The incidence of oxygen supplementation was 14 (28%) in group I as compared to 10 (20.8%) in group II (p< 0.05).

Awareness: 10 (20%) patient in Group I and 9 (16.7%) patient in Group II complained of awareness 2 hrs after surgery. Awareness was defined as recall of intraoperative events. This difference was statistically not significant.

Pain in Arm: Pain in arm due to infusion of sedative agent was found in 3 patients in Group II as against none in Group I. Nausea and vomiting was seen in 8 (16%) patients in Group I and against 4 (8.3%) patients in Group II which was statistically not significant.

Restlessness was seen in 4 (8.8%) patients in Group I as compared to 7 (14%) in Group II, which was statistically not significant.

## Discussion

When using sedative medication during regional anaesthetic technique, the anaesthesiologist attempts to titrate the drug to optimize patient comfort while maintaining cardiorespiratory stability and intact protective reflexes. The assessment of depth of sedation has been traditionally performed by observing clinical parameters such as appearance, response to voice, and pain on surgical stimulation. These parameters are qualitative and assessment of response to voice requires patient stimulation, which may itself alter depth of sedation. BIS has advantage of not requiring patient stimulation and provide a quantitative measure.

During recovery the MAP reached almost baseline in midazolam group; in propofol group, MAP remained below baseline, throughout the study period. Similar findings were reported by Hidaka *et al*^5^ in a comparison of the effects of propofol and midazolam on the cardiovascular autonomic nervous system during combined spinal epidural anaesthesia.

Arterial oxygen saturation in both the groups decreased significantly after the start of sedation. The number of patients requiring supplemental oxygen was also similar in both the groups. Almost similar results were found in Win's study^6^.

The mean recovery time (as defined by BIS >90) was significantly lower in the propofol group than the midazolam group (10.1 ±3.6 vs 18.6±6.5 min) (p=0.00). Similarly recovery times were observed by Wilson *et al*^7^ (9.2±1.5 vs 2.1±0.3 min).

As the desired sedation level was reached, the dose of anaesthetic agents was reduced and a maintenance dose of 2.2 ±0.5 mg/kg/h propofol and 0.12±0.38 mg/kg/h for midazolam had to be given. The dose for midazolam was found to be similar to Nishiyama *et al*^8^ who found that during combined spinal epidural block, midazolam 0.6 mg/kg/h was given until closing of eyes followed by midazolam 0.15 mg/ kg/h with a Ramsay sedation score of 4 along with stable haemodynamics and respiration. The incidence of side effects related to airway maintenance were similar in both the groups. However, the incidence of restlessness and pain in arm was more in propofol group but the difference was insignificant.

This study showed that though both midazolam and propofol are effective sedative agents, the time to reach effective sedation was less with propofol than midazolam and similarly the time to recovery time from sedation was lesser with propofol. Though complications were insignificant with both the drugs, propofol caused a greater fall in MABP, thus providing lesser haemodynamic stability than midazolam.
